# Risk in CNS drug discovery: focus on treatment of Alzheimer's Disease

**DOI:** 10.1186/1471-2202-9-S3-S1

**Published:** 2008-12-10

**Authors:** J Fred Pritchard

**Affiliations:** 1 Development and Regulatory Services, MDS Pharma Services, Renaissance Blvd, King of Prussia, Pennsylvania 19406, USA

## Abstract

Despite rapid progress in our understanding of disease mechanisms and an exploding list of new targets for therapeutic intervention, drug discovery and development remains a highly risky business. Understanding the risk involved requires appreciation of the differing perspectives of risk held by the various stakeholders involved in drug research. Risk can be reduced by thoughtful management of drug candidate selection, careful planning and program execution by a team of engaged experts, and disciplined decision making. Drug development is particularly challenging for treatments of neurodegenerative diseases such as Alzheimer's disease, in which translation from animal models of efficacy to human success is poor or unknown, the timelines for clinical study are long, and the markers of efficacy are still evolving. Despite this there are several therapies in clinical development that hold the promise of influencing this disease through novel and possibly synergistic mechanisms.

## Introduction

Drug discovery and development is a risky business. It can take decades to turn a new product concept into something of real value. Often commercial value is not realized until the first evidence that the drug works in patients is demonstrated, often referred to as the 'clinical proof of concept'. Thereafter, additional millions of dollars of investment are required to conduct the clinical trials necessary to substantiate safety and efficacy claims for market approval.

Approximately 11% of new drugs that enter clinical trials make it to the US market [1]. For central nervous system (CNS) drugs this rate is poorer than the average for all drugs, with only about 8% becoming available to the US public. Amplifying overall drug development risk is the falling productivity in delivering new drug entities to the marketplace [2]. This productivity decrease is occurring despite a rapidly expanding base of potentially new targets for treating disease and quantum leaps in the numbers of compounds synthesized and screened using combinatorial chemistry and high throughput methods. Since 1993, spending on research and development has increased more than threefold after adjusting for inflation, whereas the numbers of new molecular entities approved by the US Food and Drug Administration (FDA) has fallen from 30 to 50 per year in the late 1990 s to between 15 and 25 in recent years. In response, the FDA is providing leadership in re-evaluating how medical products are developed through the 'Critical Path Initiative' [3]. Several joint industry-government projects have started under this important initiative, and time will tell whether this effort will have an impact.

In the face of these brutal facts about poor success rates and dropping productivity, it seems essential that anyone participating in the business of drug discovery and development should be able to make decisions within a framework that includes practical risk assessment. This paper describes how risk is perceived (for instance, measured or defined?), what affects risk, and proven ways to manage risk, with commentary on drugs developed to treat neurodegenerative diseases.

## Defining risk

Risk (the potential for loss or injury) in drug development differs depending on which of several stakeholders you ask. These varying perspectives can be broadly collected into three views of risk: development investment risk, risk to the patient, and risk for therapeutic failure.

Development investment risk is the primary focus of company managers and owners who are weighing the investment involved against the potential for success or failure. Development investment risk depends not only on the financial resources available but also on what expertise can be engaged in the development of the drug, the regulatory requirements involved, and what other expectations exist for the product (for example, the time for development).

Risk to the patient is of interest to all of the stakeholders who are patient advocates in the process of drug development, including the pharmaceutical companies, investigators, the institutional review boards, and government regulators. Patient safety is the primary focus of FDA regulations, and approval is ultimately based on an argument of risk versus benefit or safety (injury) versus efficacy (improved health). Other drugs the patient may be taking that could interact, usually negatively, with the new drug also affect patient risk. Finally, reimbursement costs may determine who receives (or who is not willing or able to pay for) the medication.

Closely related to both of the above perspectives is risk for therapeutic failure. This is the focus of both investigators and investors. Investigators want to study drugs that have a reasonable chance of success in their patients. Investors want to back something that works and will sell, even if the chance of loss (risk) is high.

The one factor that has an impact on all three views of risk is the novelty of the product. Novel products are less predictable, thereby enhancing risk to patient, investor, and drug developer. There is no benchmark for clinical effect with new targets, making it difficult to have confidence in the animal models used in drug discovery screening. Novel drugs require more education of regulators, institutional review boards, and investigators before they feel comfortable in participating in, or giving their approval for, clinical studies. Finally, the marketplace is more leery of novel products, despite their promise of better treatment.

## Strategies for managing risk in drug discovery and development

Mitigating risk is a matter of making informed choices. Success involves selecting a good drug but then effectively managing the entire development process for this drug. The drug product will 'live or die' based on its inherent properties. However, there are many opportunities for a good drug to flounder because of poor product development decisions, inadequate investment and planning, or poor execution.

### Pick a good drug

Determining which candidate to select for further drug development from many leads can be challenging. Ideally, the selected candidate should be better than other known products that interact with the same target. If the target is novel, then there must be sufficient confidence and investment to prove the relevance of the novel target.

Part of the concept of 'translational medicine' emphasizes the need for 'valid' animal models of disease. Validity can be measured three ways. Most important in reducing overall risk is 'predictive validity', in which a drug that works in the animal will work in humans. Current animal models for Alzheimer's disease (for example, β-amyloid toxicity or rat memory impairment) have poor predictability. By comparison, those for Parkinson's disease (6OHDA in rat and MPTP in mouse or monkey) have relatively good predictive validity, but suffer because they present a much more acute pathology than the human disease. An animal model can have 'face validity' when it mimics the signs and symptoms of the human disease (for instance, memory loss in Alzheimer's disease). Finally, 'construct validity' can be claimed if the underlying pathophysiology of the animal model is the same as in the human disease (for example, β-amyloid toxicity in Alzheimer's disease, SOD1 G93A transgenic mice in amytropic lateral sclerosis).

Complementing the need for good animal models are the important new biomarker tools for measuring effect both preclinically and clinically that are emerging from an expanding universe of technologies, which include imaging, proteomics, genomics, and metabolomics. For neurodegenerative diseases, genetic testing for people who are at risk is informing the decision of who should be enrolled in certain clinical trials for potential new treatments of Alzheimer's disease, Parkinson's disease, and amytropic lateral sclerosis [4]. Magnetic resonance imaging and positron emission tomography imaging promise noninvasive ways to track many diseases [5], including the formation of neurofibrilary tangles and amyloid plaques in patients with dementia. Cellular imaging techniques are also being applied in drug discovery [6].

Over the past 15 years significant advances have been made that help to pick molecules during discovery that have the appropriate physiochemical properties to ensure acceptable manufacture and formulation, as well as predictable pharmacodynamic/pharmacokinetic/ADME (absorption, distribution, metabolism, and excretion) properties in humans. In a 2000 survey, only 12% of drugs that failed in the clinic during the preceding 10 years did so because of unacceptable pharmacodynamic/pharmacokinetic/ADME properties, as compared with nearly 50% recorded in a similar survey conducted in 1991 [1,5]. This is evidence that appropriate technology applied to selection strategies in the discovery phase does reduce risk of failure in the clinic.

In the past, 'Investigator INDs' (with IND meaning Notice of Claimed Investigational Exemption for a New Drug) were often used (and abused) as a way to get new drugs introduced into patients quickly. Investigator INDs were designed to promote clinical academic research in small numbers of patients. Often, the results of such trials have been difficult to interpret and usually provided messy information for decision making. In 2005, the FDA offered the 'Exploratory IND', as a way to give small, subtherapeutic doses of drug to humans without extensive toxicology or Chemistry, Manufacturing, and Controls documentation. This approach can offer early confirmation of half-life or bioavailability in humans, or initial evidence of efficacy, should the effect on some biomarker of the target be measurable at subtherapeutic doses. The exploratory IND offers a way to exploit emerging imaging technologies and agents (for example, positron emission tomography and magnetic resonance imaging) to assess drug effects on disease or drug distribution into the CNS. Once a drug candidate is selected, a conventional IND is required for further clinical development; nevertheless, the risk for therapeutic failure is reduced. The impact of the exploratory IND on overall costs is a matter of debate at this time.

### Have a good plan

Drug discovery and development is a process that moves through several 'go/no go' decision gates [7]. A good plan evolves by determining the key questions that must be answered before making the go/no go decision. Then, studies are designed to address these specific questions. By working backward through the process, all work done between decision gates is rationalized, wasted effort avoided, consensus built, and overall risk reduced.

### Be disciplined about decision making

At each decision gate, it is important that everyone who is key to the process understands and achieves consensus on the following questions. What is the decision process (directive, democratic, or consensus)? Who decides, consults, or is informed of the decision? What are the choices? By studying scenarios ahead of time, one builds confidence in the decision strategy. Often, this is framed in terms of criteria for deciding, examples being the successful use of the 'target product profile' in drug development.

### Involve the right people

Successful modern drug development is managed by a team of committed people who represent needed expertise and are led by a 'champion' for the project. The leader educates decision makers and ensures priority and resources for assigned work. Successful teams look for synergy in their efforts built on mutual respect.

### Execute well

Effective execution involves good project management skills to ensure that work is done on time and on budget. Moreover, every successful drug had a team that reacted to unforeseen events in a timely and reasoned way through open dialog that started with contingency planning.

## Challenges facing development of CNS drugs

Compared with other therapeutic categories, CNS drugs – particularly those targeted at neurodegenerative disease – face certain unique challenges that add risk.

### Evolving translational research

There are few predictive animal models of human neurodegenerative diseases, including Alzheimer's disease. Adding to the complexity is that no single disease mechanism has emerged as the dominant driver for the emergence and progression of this disease. Recent legislation and agreements requiring public disclosure of clinical trial results, both positive and negative, should improve access to human data. Extensive human information is required before we know the translational power of animal models to human experience. Over the next few years we will learn from patients suffering from Alzheimer's type dementia whether drugs that reduce β amyloid build-up or block its neurotoxicity will outperform those that stimulate neurotransmitter function, those that reduce inflammation, and those that have neurotropic properties.

### Changing standards of care

It is likely that future therapies will involve a combination of products that attack different features of the disease and will hopefully work synergistically in providing relief from the progression of Alzheimer's disease. This approach is already being used as a standard of care in many areas of the USA, where a cholinesterase inhibitor is being prescribed together with memantine under the belief that two therapies will be better than one. So far, the practice is limited by tolerance issues in some patients, as well as the patient's ability to pay for more than one pharmaceutical treatment for their disease. Nevertheless, multidrug therapy creates another challenge in designing early clinical studies to determine whether new and novel therapies are effective and safe. Most patients (and their care givers) will not wish to be randomized to the risk for being treated with no or limited treatment for long periods of time (3 months to 1 year), which is a defining requirement for a statistically credible control group. Therefore, we are forced to consider add-on trial designs. By their nature, phase II studies involve a relatively small number of patients (50 to 400) because it is usually not prudent to invest in large clinical trials and drug manufacture without some signal of efficacy. It can be very challenging under these circumstances to design a study with sufficient statistical power to inform good decision making. In addition, add-on designs place greater weight on early consideration of potential drug-drug interactions. Fortunately, there has been good progress in recent years in the strategies and science surrounding early predication of how one drug might affect the metabolism of another.

### Biomarker development

Developing relevant biomarkers of effect is one way to work around these issues and obtain clinical proof-of-concept information more quickly. Unfortunately, no biochemical markers in human plasma, serum, blood, or urine have emerged as broadly accepted predictors of disease progression or as sensitive indicators of the effect of therapeutic intervention on specific pharmacologic targets. Brain imaging technology holds the most hope and is the focus of considerable current clinical research effort.

Often, CNS diseases progress in severity beyond a point of effective intervention. However, people with mild disease are difficult to identify. There is strong commercial and academic interest in the development of early biomarkers that can diagnose the presence of mild disease even before behavioral and memory symptoms appear. Conversely, patients with severe or refractory disease make poor trial candidates.

### Drug delivery to the brain

Delivering active drug past the blood-brain barrier can be a challenge. Unless one has a long-acting drug product, parenteral routes of administration would not be practical in a typical Alzheimer's patient setting. A once-a-day oral product that distributes readily to the site of action (CNS) is the ideal; most currently marketed drugs for treating Alzheimer's symptoms possess these features.

### Competition for study participants

There are several new therapies that are currently in early clinical trials as well as continued testing to optimize use of currently marketed agents. However, despite an expanding patient base, the intensity of clinical research does mean that different studies and therapies are competing for an increasingly limited pool of patients. Collaborative study designs involving two or three new drug products may be the only practical solution that could address the efficiencies and ethics of performing large clinical trials in this area.

Unlike most other clinical drug development programs, the study of treatments for neurodegenerative diseases involves both the patient and their care giver. The additional challenge of relying on two different perspectives in therapeutic assessment and study management cannot be understated.

## Conclusion

Risk management involves understanding the different perspectives among stakeholders and making informed choices. Keys to success are picking a good drug (although the models and biomarkers may be less than 'good') and then managing its development well. Modern drug discovery techniques, properly applied, reduce the risk for failure in the clinic and provide hope for better future therapies for neurodegenerative disease.

## List of abbreviations used

ADME: administration, distribution, metabolism, and excretion; CNS: central nervous system; IND: Notice of Claimed Investigational Exemption for a New Drug.

## Competing interests

The author is employed by a provider of consulting, laboratory, and clinical services to the pharmaceutical industry. The author owns stock in MDS Inc. and several pharmaceutical companies; however, publication of this article is not believed to influence financial performance of these companies. This author presented this paper by invitation at the 2007 Drug Discovery Conference sponsored by the Alzheimer's Drug Discovery Foundation and the Institute for the Study of Aging, held in New York City (5 to 6 February 2007).
